# Low-affinity Nerve Growth Factor Receptor (CD271) Heterogeneous Expression in Adult and Fetal Mesenchymal Stromal Cells

**DOI:** 10.1038/s41598-018-27587-8

**Published:** 2018-06-18

**Authors:** Mario Barilani, Federica Banfi, Silvia Sironi, Enrico Ragni, Salomé Guillaumin, Francesca Polveraccio, Lorenzo Rosso, Monica Moro, Giuseppe Astori, Michela Pozzobon, Lorenza Lazzari

**Affiliations:** 1 Laboratory of Regenerative Medicine – Cell Factory, Fondazione IRCCS Ca’ Granda Ospedale Maggiore Policlinico, 20122 Milano, Italy; 2 Regenerative, Modular & Developmental Engineering Laboratory (REMODEL), Biomedical Sciences Building, National University of Ireland Galway (NUI Galway), H91 CF50 Galway, Ireland; 3 Science Foundation Ireland (SFI) Centre for Research in Medical Devices (CÚRAM), Biomedical Sciences Building, National University of Ireland Galway (NUI Galway), H91 CF50 Galway, Ireland; 4 Thoracic surgery and lung transplantation Unit, Fondazione IRCCS Ca’ Granda Ospedale Maggiore Policlinico, 20122 Milano, Italy; 5 University of Milan, 20122 Milano, Italy; 6 INGM, National Institute of Molecular Genetics “Romeo ed Enrica Invernizzi”, 20122 Milan, Italy; 7 Advanced Cellular Therapy Laboratory - Hematology Unit, S. Bortolo Hospital - ULSS 6, Contra’ San Francesco 41, 36100 Vicenza, Italy; 8 Stem Cells and Regenerative Medicine Lab., Women’s and Children’s Health Dept., University of Padova, Via Giustiniani 3, 35128 Padova, Italy; 9 Foundation Institute of Pediatric Research “Città della Speranza”, Corso Stati Uniti 4, 35127 Padova, Italy

## Abstract

Human multipotent mesenchymal stromal cells (MSC) are isolated from a plethora of tissue sources for cell therapy purposes. In 2006, the International Society for Cellular Therapy (ISCT) published minimal guidelines to define MSC identity. Nevertheless, many independent studies demonstrated that cells meeting the ISCT criteria possessed heterogeneous phenotypes and functionalities, heavily influenced by culture conditions. In this study, human MSC derived from many adult (bone marrow and adipose tissue) or fetal (cord blood, Wharton’s jelly, umbilical cord perivascular compartment and amniotic fluid) tissues were investigated. Their immunophenotype was analyzed to define consistent source-specific markers by extensive flow cytometry analysis and real-time qRT-PCR. CD271^+^ subpopulations were detected in adult MSC, whereas NG2 was significantly more expressed in fetal MSC but failed validation on independent samples coming from an external laboratory. The highest number of CD271^+^ adult MSC were detected soon after isolation in serum-based culture conditions. Furthermore, heterogeneous percentages of CD271 expression were found in platelet lysate-based or serum-free culture conditions. Finally, CD271^+^ adult MSC showed high clonogenic and osteogenic properties as compared to CD271^−^ cells. To conclude, in this phenotype-function correlation study CD271^+^ subpopulation confers heterogeneity on adult MSC, confirming the need of more specific markers to address MSC properties.

## Introduction

Over the past 15 years, the amount of human multipotent mesenchymal stromal cell (hMSC) research has grown exponentially. In addition to bone marrow^1^, a plethora of MSC tissue sources have been uncovered, suggesting that MSC could be found in virtually any vascularized tissue of the body^2,3^. This finding lead to the identification of pericytes, PDGFRβ^+^/CD146^+^/NG2^+^/CD34^−^/CD31^−^ mural cells that wrap around blood microvessels, as the *in vivo* progenitors of *in vitro* isolated and cultured MSC. Pericytes can differentiate into adipocytes, chondrocytes, osteoblasts, and myocytes *in vitro*. An updated version of this theory claims that the genuine native MSC progenitors may be cells found in the outermost layer of arterial adventitia^4^. MSC are subdivided into adult and fetal cells, depending on their tissue of origin. Adult MSC include those derived from bone marrow (BMMSC)^5^ and adipose tissue (ADMSC)^6^, whereas fetal MSC originate from amniotic fluid (AFC)^7^, cord blood (CBMSC)^8–10^, or the perivascular compartment (PVC) or Wharton’s jelly (WJMSC) of the umbilical cord^11^. More recently, MSC were also derived from pluripotent stem cells (PSC), such as embryonic stem cells (ESC)^12,13^ or induced PSC (iPSC)^14,15^. PSC-derived MSC were termed PD-MSC, while their best isolation strategy and functional properties are still under investigation^16^.

The enormous focus on MSC research has led to the development of a wide but controversial and chaotic field of literature. The most important issue in MSC research regards the heterogeneity of MSC derived from different tissue sources, donors of different ages, as well as inter- and intra-donor variability^17^. Furthermore, culture conditions heavily influence MSC behavior and features *in vitro*^18,19^. The widespread lack of culture condition homogeneity between laboratories has made it difficult to reach unequivocal consensus even on basic MSC properties, leading to discrepancies at the basic research level and adding biases to the evaluation of apparently contrasting stem cell-based clinical study outcomes^20,21^.

In 2006, the International Society for Cellular Therapy (ISCT) provided a minimal set of standard criteria to define MSC identity^22^, which included physical properties (ability to adhere to the surface of uncoated plastic surfaces), immunophenotypic properties (positivity for CD105, CD73, and CD90 and negativity for CD45, CD34, CD14 or CD11b, CD79a or CD19, HLA-DR), and functional properties (potential to differentiate into osteoblasts, adipocytes, and chondrocytes *in vitro*). These criteria have been extremely helpful to provide a common ground and set of accepted parameters for the MSC field. Nonetheless, given the aforementioned issues, these standard criteria need to be readdressed.

The possible benefits of therapeutic applications of hPSC have led to high expectations, as underlined by the number of ongoing clinical trials (21 trials, as reported by ClinicalTrial.gov with “embryonic stem cell” as search terms; 2018/04/03). However, hMSC are still the most employed stem cell type in the regenerative medicine field, with 3,327 ongoing clinical trials (“MSC OR multipotent mesenchymal stromal cell OR mesenchymal stem cell” search terms; 2018/04/03). In spite of the huge interest raised by MSC, the extensive number of potential MSC sources, isolation protocols, culture conditions, and prospective or a posteriori characterization assays have contributed to the inconsistency and contrasting results concerning the definition of MSC identity and functional properties^23–25^. Several reports have compared the phenotype of MSC isolated from different sources although those studies limited their research to only a few tissue sources. There is an urgent need to deepen knowledge concerning markers of *in vitro* cultured MSC from different sources, as more specific identity-defining determinants. Furthermore, the 2006 position paper by ISCT, which proposed minimal criteria for MSC identity definition, would benefit from a deeper characterization of MSC features. Therefore, we performed extensive analyses on MSC from the major adult and fetal sources and on human skin fibroblasts (HSF, as stromal non-stem control); PD-MSC were also tested. We carefully maintained homogeneous culture conditions and cell manipulations to remove any possible bias from our study.

## Results

### Adult and fetal MSC share similar morphology and clonogenic potential

**Figure 1:**
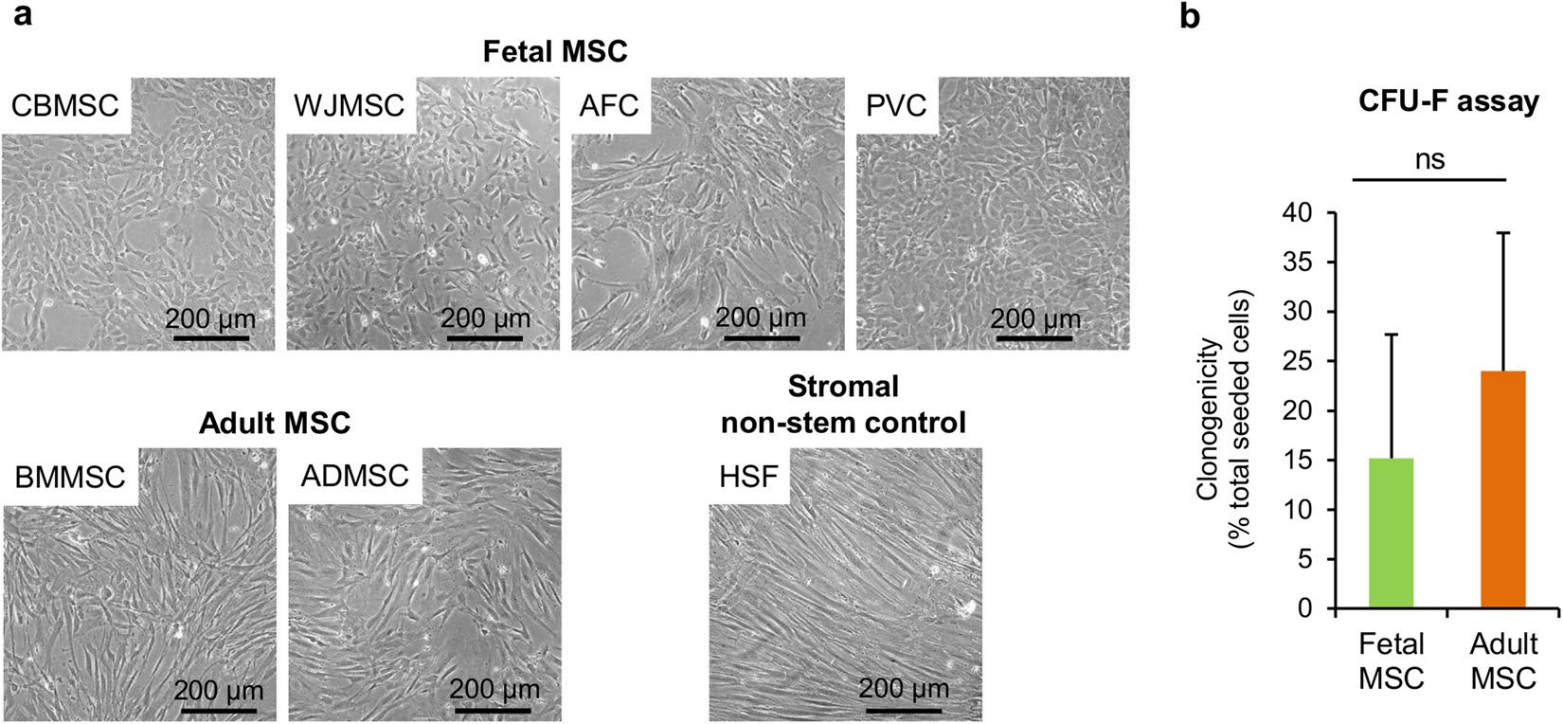
Fetal and adult multipotent mesenchymal stromal cell (MSC) morphology. (**a**) Representative bright-field microscopy images of cultured MSC isolated from fetal and adult tissue sources, and of human skin fibroblasts (HSF). Fetal and adult MSC clonogenic potential. (**b**) The histogram shows the percentage of cells with clonogenic capability under low-density seeding conditions for fetal and adult MSC (*n* = 3 for each cell population). Statistical analysis was by non-parametric two-tailed Mann-Whitney-Wilcoxon test (*P* = 0.5333); ns, statistically not significant difference. ADMSC, adipose tissue MSC; BMMSC, bone marrow MSC; CBMSC, cord blood MSC; WJMSC, Wharton’s jelly MSC; PVC, perivascular cell; AFC, amniotic fluid cell.

No major differences in cell morphology were found among the cells, although adult MSC showed a more fibroblastic-like shape compared to the generally more compact and less elongated morphology of fetal MSC (Fig. 1a). MSC were also tested for their clonogenic properties under low-density seeding conditions (Colony Forming Unit-Fibroblasts (CFU-F) assay). All MSC types retained the potential to generate colonies, and no statistically significant differences were found between fetal and adult MSC, as shown in Fig. 1b.

### Basic and extended immunophenotype of fetal and adult MSC under controlled culture conditions

Immunophenotypes of MSC and HSF were addressed by extensive flow cytometry analysis to characterize stromal cells at the protein level and define specific immunophenotypic panels for different MSC sources. The classic surface markers used to define MSC were investigated, and no differences were found comparing both stem and non-stem stromal cells or MSC derived from different tissues (Table 1).

**Table 1:** ISCT surface marker panel.

*Surface markers*	HSF *(mean* ± s.d.)	ADMSC *(mean* ± s.d.)	BMMSC *(mean* ± s.d.)	CBMSC *(mean* ± s.d.)	WJMSC *(mean* ± s.d.)	PVC *(mean* ± s.d.)	AFC *(mean* ± s.d.)
*CD90*	99% ± 1	99.5 ± 0.6%	100 ± 0%	100 ± 0%	100 ± 0%	100 ± 0%	98 ± 1%
*CD105*	92% ± 2	98 ± 1.2%	97.3 ± 2.2%	100 ± 0%	100 ± 0%	100 ± 0%	98 ± 2%
*CD73*	86% ± 4	99.9 ± 0.1%	99.9 ± 0.2%	95 ± 2%	100 ± 0%	99 ± 1%	99 ± 1%
*CD271*	3.2 ± 2.6%	8.4 ± 4.6%	3.7 ± 2.2%	<0.5%	<0.5%	<0.5%	<0.5%
*CD90/CD271*	98 ± 1.5%	99 ± 1.1%	100 ± 0%	—	—	—	—
*CD73/CD271*	100 ± 0%	100 ± 0%	99.9 ± 0.1%	—	—	—	—
*CD45*	<1%	<1%	<1%	<1%	<1%	<1%	<1%
*CD34*	<1%	<1%	<1%	<1%	<1%	<1%	<1%
*CD14*	<1%	<1%	<1%	<1%	<1%	<1%	<1%

Alternative surface markers were selected from the existing literature, given their ability to identify MSC subpopulations with different stemness properties; these markers were CD271, NG2, CD56, PDGFRβ, and SSEA4. Marked differences were found between adult and fetal MSC for these surface markers, with adult MSC showing high phenotypic similarity to HSF for some of them (Supplementary Fig. 1).

**Figure 2:**
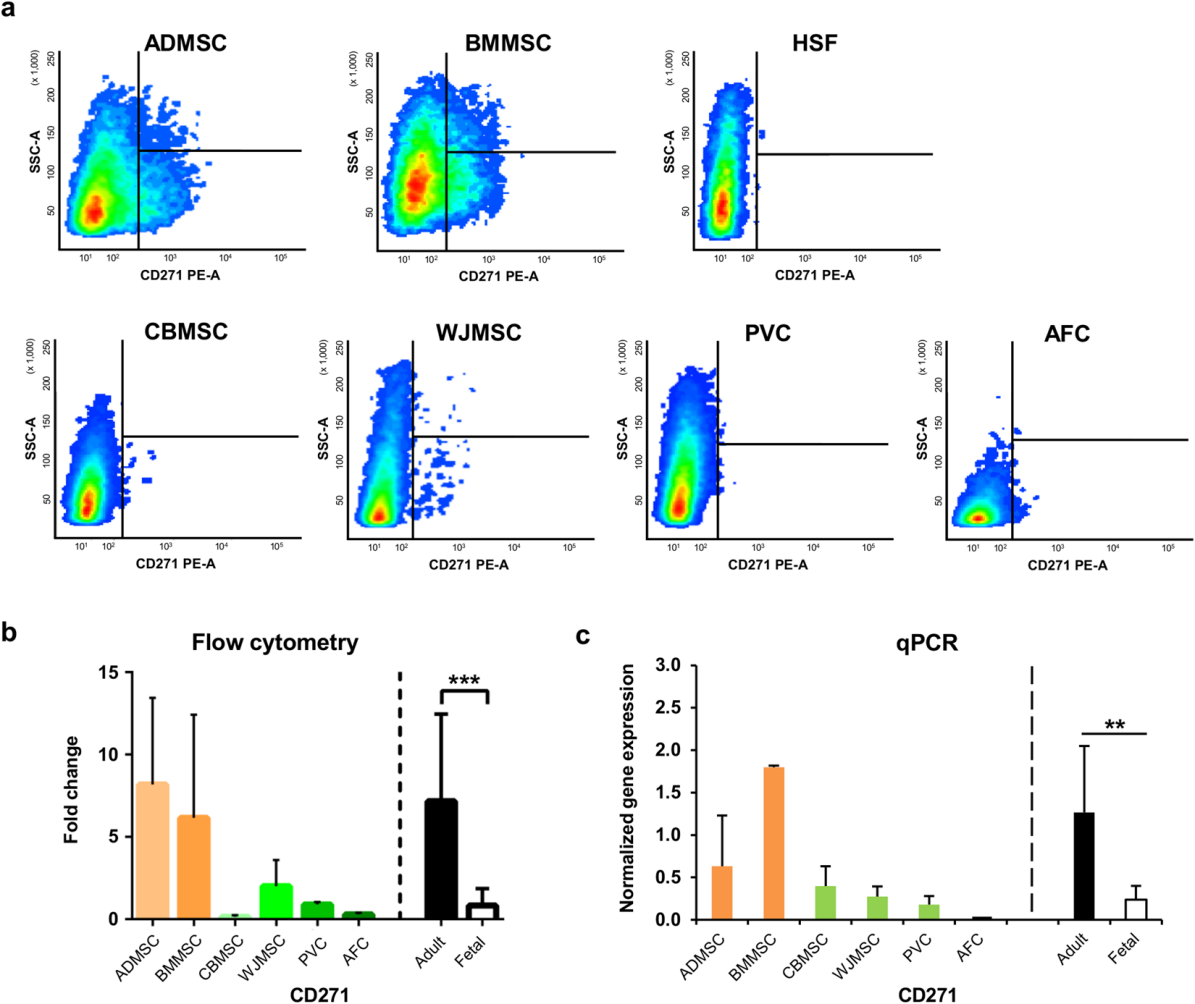
Adult multipotent mesenchymal stromal cells (MSC) exhibit CD271-positive subpopulation. (**a**) Representative dot plots of CD271 expression by adult and fetal MSC and of human skin fibroblast (HSF). (**b**) CD271 surface marker expression and (**c**) gene expression, both normalized to stromal non-stem control (HSF). Mean and standard deviation for single MSC types (*n* = 3) or for adult (*n* = 6) *vs*. fetal (*n* = 12) MSC groups (separated by dotted line). Statistical analysis was by non-parametric two-tailed Mann-Whitney-Wilcoxon test (*P* = 0.0004) or by Kolmogorov-Smirnov normality test (α = 0.05), followed by unpaired two-tailed *t*-test (*P* = 0.012); **P < 0.01, ***P < 0.001. ADMSC, adipose tissue MSC; BMMSC, bone marrow MSC; CBMSC, cord blood MSC; WJMSC, Wharton’s jelly MSC; PVC, perivascular cell; AFC, amniotic fluid cell; adult, MSC from adult sources (black bar); fetal, MSC from fetal sources (white bar).

**Figure 3:**
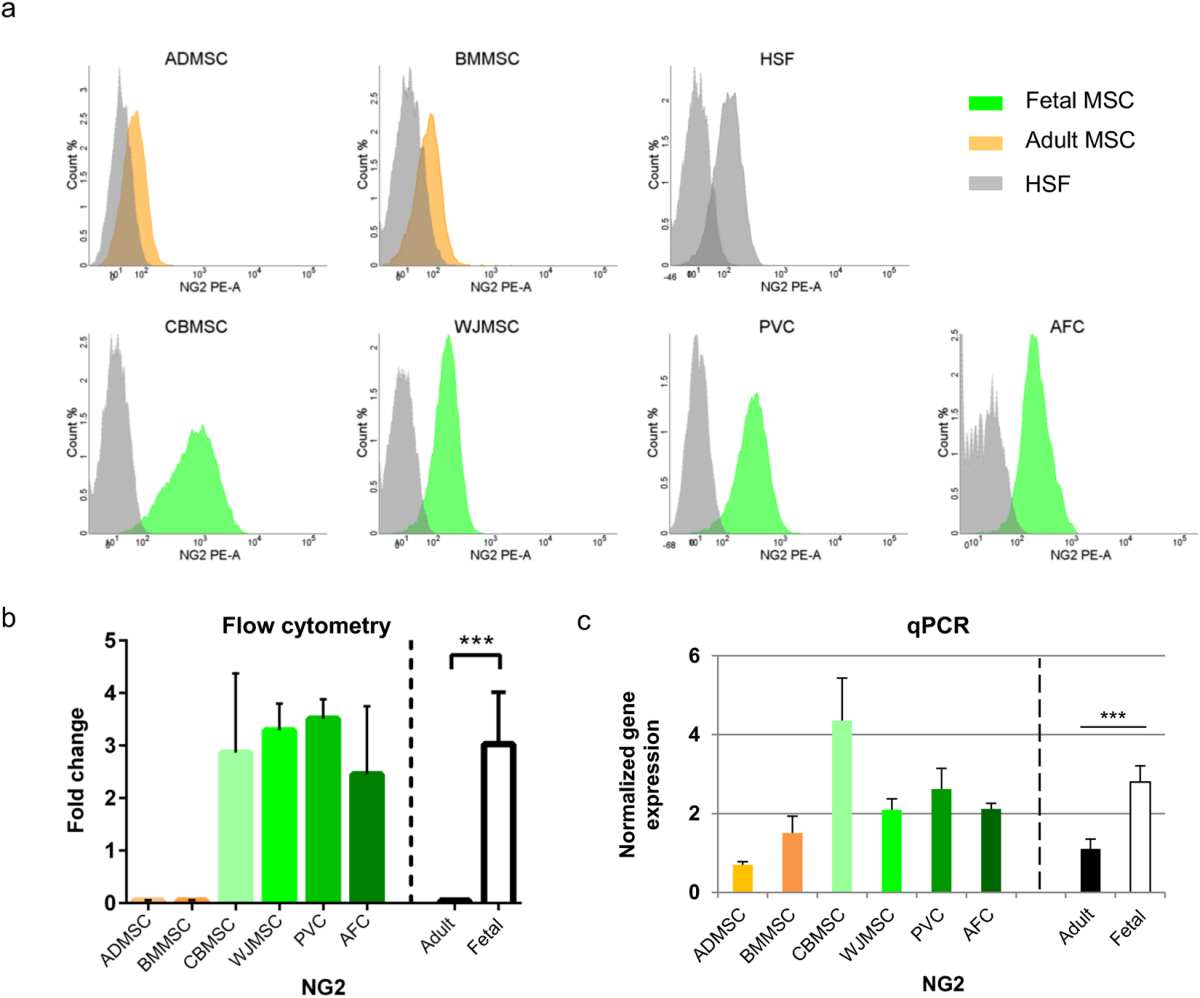
Fetal multipotent mesenchymal stromal cells (MSC) show high positivity for NG2. (**a**) Representative histograms of NG2 expression by adult and fetal MSC and of human skin fibroblast (HSF). (**b**) NG2 surface marker expression and (**c**) gene expression, both normalized to stromal non-stem control (HSF). Mean and standard deviation for single MSC types (*n* = 3) or for adult (*n* = 6) *vs*. fetal (*n* = 12) MSC groups (separated by dotted line). Statistical analyses were by non-parametric two-tailed Mann-Whitney-Wilcoxon test (b, *P* = 0.001), or by Kolmogorov-Smirnov normality test (α = 0.05) followed by unpaired two-tailed *t*-test (c, *P* = 0.0001); ***P < 0.001. ADMSC, adipose tissue MSC; BMMSC, bone marrow MSC; CBMSC, cord blood MSC; WJMSC, Wharton’s jelly MSC; PVC, perivascular cell; AFC, amniotic fluid cell; adult, MSC from adult sources (black bar); fetal, MSC from fetal sources (white bar).

Adult MSC showed a cell subpopulation positive for low-affinity nerve growth factor receptor (CD271), which was absent or not consistently detectable in fetal MSC and in control HSF (Fig. 2a and Supplementary Fig. 1). Adult MSC were significantly enriched (*P* < 0.0001) in these CD271-positive cells (Fig. 2b), compared to fetal MSC. The same statistically significant trend was also observed at the mRNA level (Fig. 2c). In addition, CD271^+^ adult MSC were highly positive also for CD90 and CD73 (Table 1). Increased levels of the pericytic surface marker NG2 was found for all fetal MSC (Mean Fluorescence Intensity (MFI) ratio 20.7 ± 14.2) compared to adult MSC (MFI ratio 3.8 ± 0.8) (Fig. 3a); HSF control showed a 6.8 ± 0.6 MFI ratio. Fetal MSC showed significantly high (*P* < 0.001) NG2 expression (Fig. 3b), compared to adult MSC. This difference was confirmed to be statistically significant (*P* < 0.001) also at the mRNA level (Fig. 3c).

**Figure 4:**
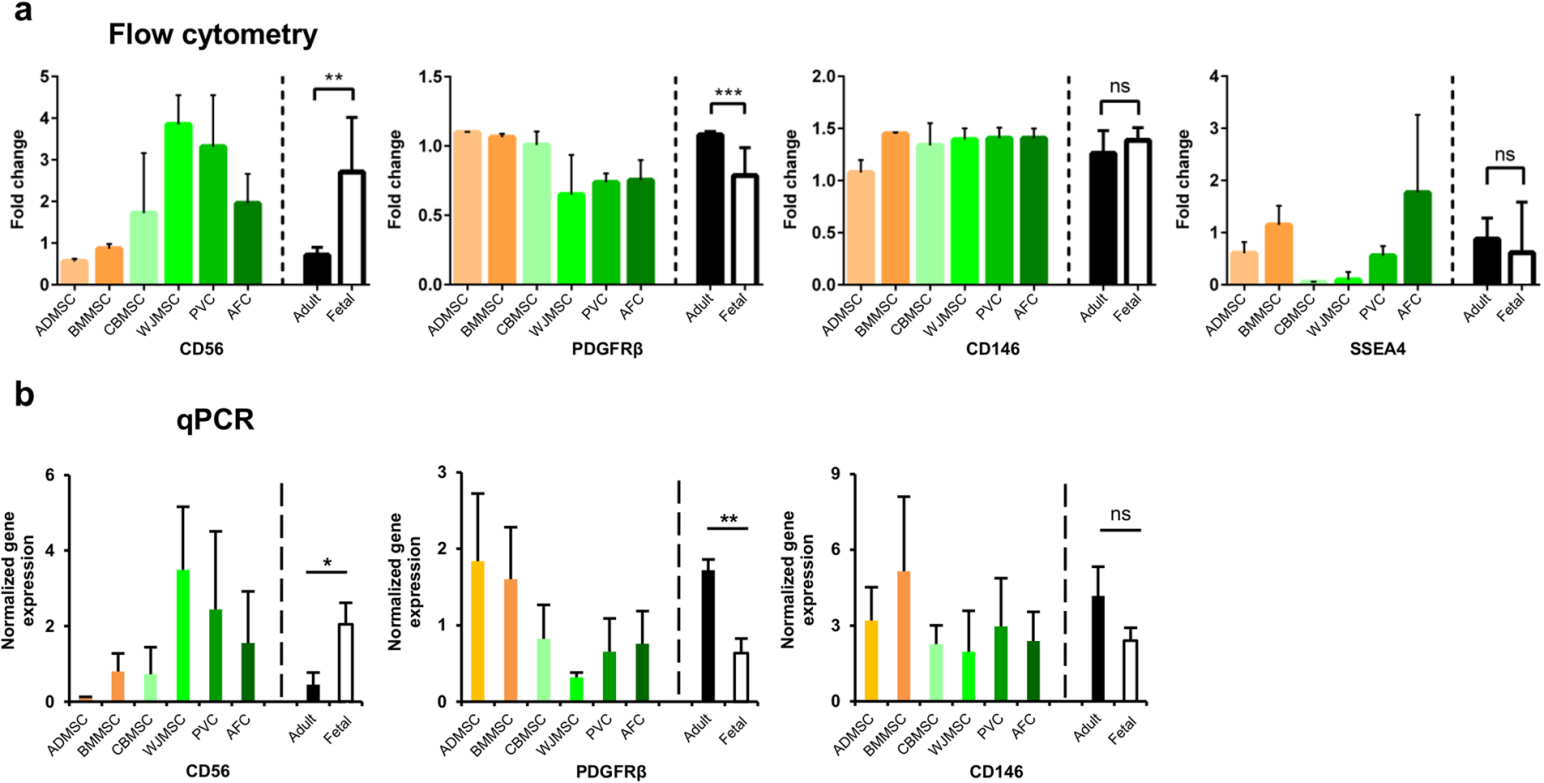
Fetal and adult multipotent mesenchymal stromal cells (MSC) expression of progenitor- and stemness-associated surface markers. (**a**) Representative histograms of surface marker expression (CD56, PDGFRβ, CD146 and SSEA4) and (**b**) gene expression (CD56, PDGFRβ, CD146; not applicable for SSEA4), both normalized to stromal non-stem control (human skin fibroblasts, HSF). Mean and standard deviation for single MSC types (*n* = 3) or for adult (*n* = 6) *vs*. fetal (*n* = 12) MSC groups (separated by dotted line). Statistical analysis was by Kolmogorov-Smirnov normality test (α = 0.05), followed by unpaired two-tailed *t*-test for CD56 (a, *P* = 0.021; b, *P* = 0.0397), CD146 (b, *P* = 0.598) and SSEA4 (a, *P* = 0.0775), by non-parametric two-tailed Mann-Whitney-Wilcoxon test for CD146 (a, *P* = 0.0976), PDGFRβ (a, *P* = 0.0007; b, *P* = 0.0032); *P < 0.05, **P < 0.01, ***P < 0.001; ns, statistically not significant difference. ADMSC, adipose tissue MSC; BMMSC, bone marrow MSC; CBMSC, cord blood MSC; WJMSC, Wharton’s jelly MSC; PVC, perivascular cell; AFC, amniotic fluid cell; adult, MSC from adult sources (black bar); fetal, MSC from fetal sources (white bar).

In addition, stemness-associated CD56 was found to be significantly (*P* < 0.01) more expressed in fetal MSC than in adult MSC. In contrast, pericytic marker PDGFRβ was found to be significantly (*P* < 0.001) more expressed in adult MSC than in fetal MSC (Fig. 4a). Both markers showed the same significant (*P* < 0.05 the former; *P* < 0.01 the latter) differences also at the transcriptional level (Fig. 4b). SSEA4 was not addressed by gene expression analysis in reason of its glycosphingolypid identity.

No significant differences were found between adult and fetal MSC for CD146 and SSEA4 (Fig. 4a), but also between MSC and HSF (Supplementary Fig. 1), notwithstanding their proposed role as stem cell markers. In detail, ADMSC and HSF showed similarly decreased expression levels of CD146 compared to MSC from all other sources. Also at the transcriptional level, no significant difference in CD146 gene expression was observed (Fig. 4b). Regarding SSEA4, MSC from all sources showed highly heterogeneous expression, even though the majority of fetal MSC, with the exception of AFC, showed a reduced expression compared to adult MSC.

Finally, when the same flow cytometry analysis was performed at different passages (P3 and P5) for BMMSC and CBMSC as representative adult and fetal MSC populations, no differences of the immunophenotype were observed (data not shown).

### Fetal and adult MSC immunophenotype is reminiscent of the tissue of origin

**Figure 5:**
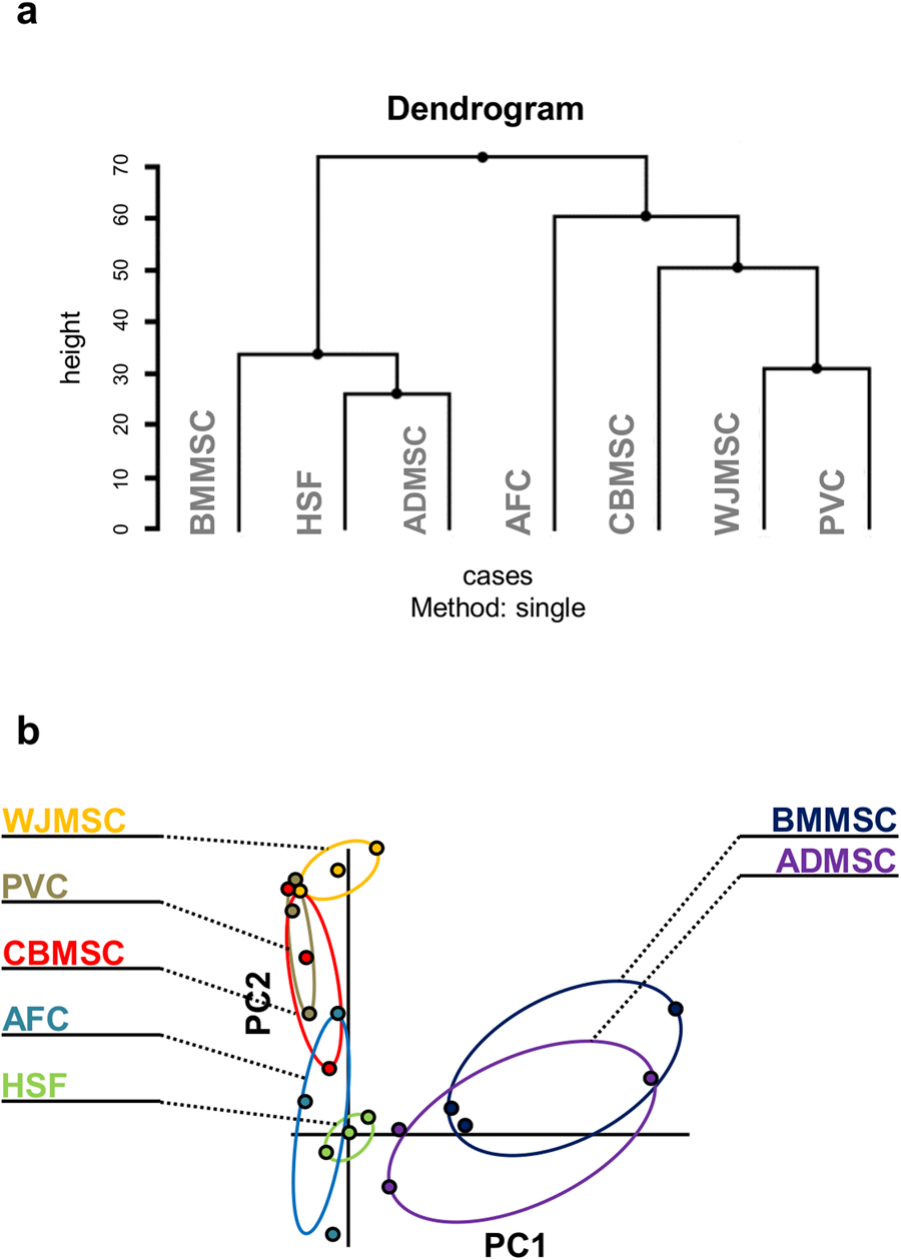
Hierarchical clustering of multipotent mesenchymal stromal cell (MSC) immunophenotypes separates fetal and adult harvest. (**a**) Dendrogram generated by single linkage clustering. Height represents relative distance between clusters. (**b**) Principal component (PC) analysis showing grouping of MSC by source, centered on human skin fibroblasts (HSF) as controls, relative to PC1 and PC2 variables. ADMSC, adipose tissue MSC; BMMSC, bone marrow MSC; CBMSC, cord blood MSC; WJMSC, Wharton’s jelly MSC; PVC, perivascular cell; AFC, amniotic fluid cell.

Cluster analysis was performed to address whether the flow cytometry data were sufficient to segregate MSC based on their tissue source. Interestingly, the implemented immunophenotypic panel was able to group and discriminate adult and fetal MSC (Fig. 5a). Also in this analysis, ADMSC preferentially grouped with HSF. On the other hand, AFC were the most distant from the other more compact fetal MSC, as showed by its segregation under an independent node of the hierarchical clustering dendrogram.

Principal component analysis (PCA) was also applied to the flow cytometry data, and the results were plotted by using HSF as centered control (Fig. 5b). These results confirmed the hierarchical clustering showing a clear separation between adult and fetal MSC, with the adult group showing higher heterogeneity.

### Adult MSC harbor a CD271^+^ subpopulation

**Figure 6:**
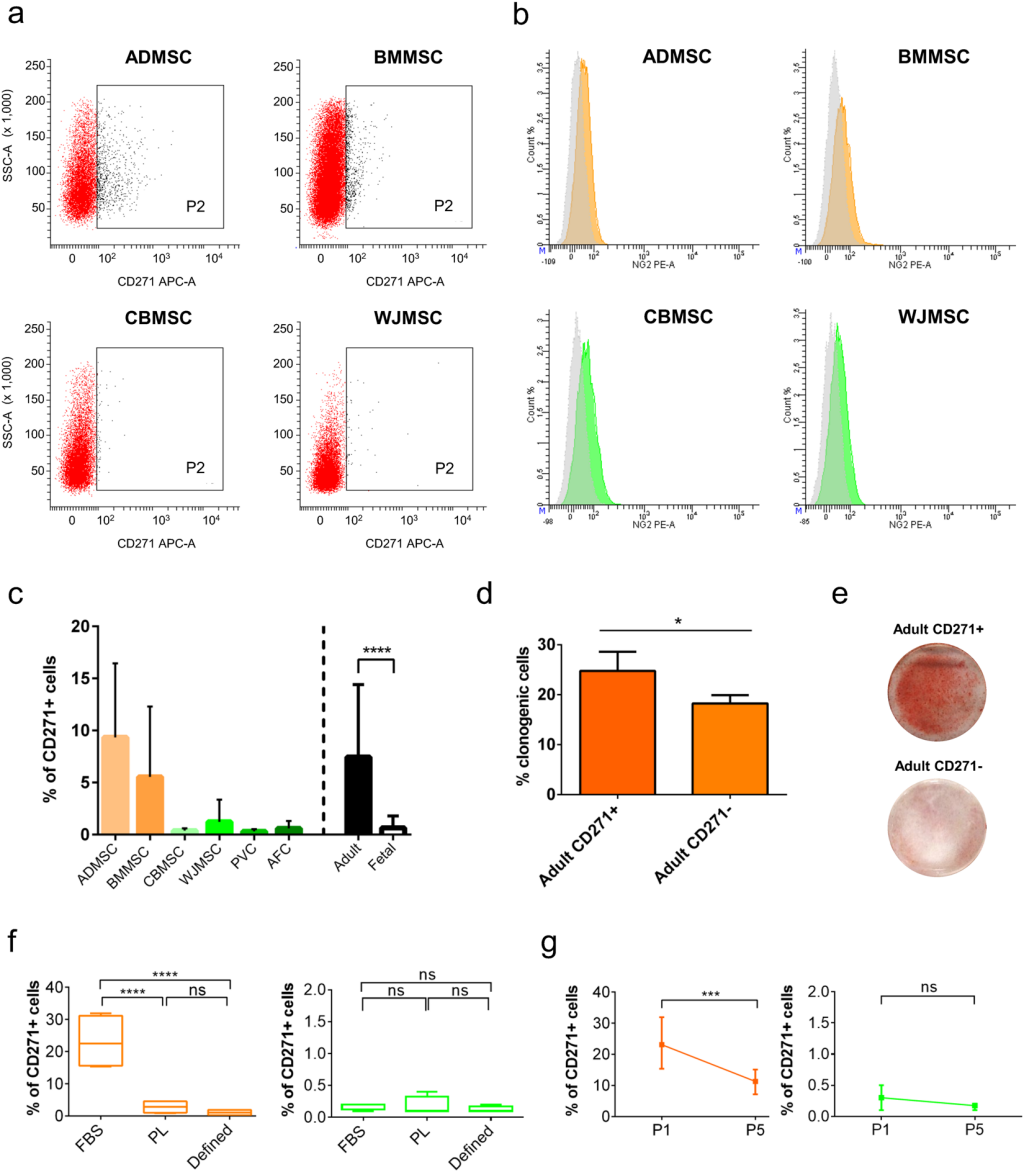
Adult MSC harbor a CD271^+^ subpopulation. (**a**) Representative dot plots showing CD271 expression in additional MSC populations isolated by an independent laboratory. (**b**) Representative histograms showing NG2 expression in additional MSC populations isolated by an independent laboratory. (**c**) Validation of CD271 expression in *n* = 8 samples for each MSC source (adult MSC, *n* = 16; fetal MSC, *n* = 32). (**d**) Percentage of colony forming unit-fibroblasts (CFU-Fs) in sorted CD271^+^ (adult CD271^+^) and CD271^−^ (adult CD271^−^) adult MSC. (**e**) Representative cultures of sorted CD271^+^ (adult CD271^+^) and CD271^−^ (adult CD271^−^) adult MSC, which received osteogenic stimuli. (**f**) CD271 expression in adult and fetal MSC under different culture conditions. (**g**) CD271 expression in adult and fetal MSC at early and late passages. Statistical analysis was by unpaired two-sided Mann-Whitney-Wilcoxon test for c (*P* = 0.0001) and g (Adult P1 vs P5, *P* = 0.0286; fetal P1 vs P5, *P* = 0.5143), by unpaired one-sided Mann-Whitney-Wilcoxon test for d (*P* = 0.0429), by two-way ANOVA (α = 0.05) followed by Tukey’s multiple comparisons test for f (FBS vs PL, *P* < 0.0001; FBS vs defined, *P* < 0.0001; PL vs defined, *P* > 0.05); *P < 0.05, ****P < 0.0001; ns, statistically not significant difference. ADMSC, adipose tissue MSC; BMMSC, bone marrow MSC; CBMSC, cord blood MSC; WJMSC, Wharton’s jelly MSC; adult, adult MSC; fetal, fetal MSC.

CD271 and NG2 were the most promising antigens showing marked differences in our experimental conditions between fetal and adult MSC. To validate this result, the immunophenotypes and gene expression levels of eight additional MSC samples, isolated and expanded by an independent laboratory with the same culture conditions applied for the previous samples, were investigated. Analyses were performed on ADMSC, BMMSC, CBMSC and WJMSC. Concerning CD271, a significantly higher (*P* = 0.0286) CD271^+^ subpopulation was observed in all adult MSC (3.8 ± 2.3%, *n* = 4) but were absent in fetal MSC (0.6 ± 0.1%, *n* = 4), strongly confirming our previous results (Fig. 6a). In contrast, NG2 was expressed at very low levels in adult MSC (2.5 ± 0.9 MFI ratio), but also in fetal MSC (2.1 ± 0.8 MFI ratio), failing the validation (Fig. 6b). To make the CD271 result more robust, its expression was addressed in a higher number of samples (*n* = 8 for each MSC type; adult MSC *n* = 16, fetal MSC *n* = 32). Figure 6c once again shows the strongly significant difference in CD271 expression between adult and fetal MSC. The immunophenotype of PD-MSC (*n* = 3) was also addressed, resulting in high CD90^+^/CD73^+^ (99.6 ± 0.7%) and low CD105^+^/CD73^+^ (10.6 ± 7.5%) expression; CD271 was weakly expressed (1.6 ± 1%). To explore a functional relevance for the adult MSC CD271^+^ subpopulation, a clonogenic (CFU-F) assay was performed on CD271^+^ sorted cells. Intriguingly, CD271^+^ adult MSC showed a significant high percentage of colony-forming cells compared to CD271^−^ adult MSC (Fig. 6d). Sorted cells were also induced to differentiate into osteocytes. As shown in Fig. 6e, mineralization was observed in CD271^+^ adult MSC cultures and not in the negative counterpart. Moreover, to check the antigen stability in the presence of different supplements, CD271 expression was addressed in adult and fetal MSC cultured in different media: αMEM supplemented with 20% FBS (FBS) or with 5% PL (PL), and a serum-free xeno-free chemically-defined GMP-grade medium (defined). In adult MSC CD271 was more expressed in the FBS condition than in PL and defined ones, while in fetal MSC the CD271^+^ cells was <0.5% for all conditions (Fig. 6f). Finally, we checked stability of CD271 expression along passages. As shown in Fig. 6g, a decreasing trend was observed for adult MSC but not for fetal MSC.

## Discussion

In the present study, we aimed to answer the following research question: are there surface markers able to efficiently discriminate tissue-specific MSC *in vitro*, unbiased by the applied culture conditions?

Initially, we addressed the expressions of classic mesenchymal surface proteins (CD90, CD105, CD73). In accordance with previous reports, we found that MSC shared expressions of these proteins with HSF, leading to questions about their utility as specific MSC markers^26,27^. We therefore concluded that these antigens, commonly recognized as MSC specific markers, are instead to be more appropriately considered as stromal cell markers. Therefore, we moved to the evaluation of alternative antigens, recently proposed as stemness-associated MSC markers. Among this new set of surface markers (NG2, CD56, CD271, CD146, PDGFRβ, and SSEA4), we finally highlighted statistically significant differences between adult and fetal MSC and HSF.

In particular, two important stemness-associated surface markers, NG2 and CD56, were strongly upregulated in fetal compared to adult MSC, when cultured under controlled conditions. Notwithstanding their typical expression in neural tissues, our group previously demonstrated NG2 specific expression in pericytes *in vitro* and *in vivo*, and this has a relevant meaning for MSC, because pericytes are considered to be the *in vivo* progenitors of *in vitro* MSC^2^.

Surprisingly, other pericytic markers, PDGFRβ and CD146, were widely expressed among all stromal cell types, including HSF. Yet, a slight reduction of CD146 expression in ADMSC to levels similar to HSF may hint at a more differentiated state^28^.

Focusing on SSEA4, it is a well-known embryonic stem cell marker that has been proposed as a marker to differentiate genuine bone marrow-derived MSC. Again, we surprisingly detected this antigen in almost all stromal cells, with HSF showing expression levels comparable to adult MSC, while fetal MSC showed heterogeneous expression profiles. Yet, we recommend to carefully evaluate this surface marker, because SSEA4 expression in cord blood hematopoietic stem cells (CB-HSCs) was recently suggested to be an artifact due to *in vitro* culture. Fetal calf serum contains globoseries glycosphingolipids, which can be recognized by SSEA4 antibodies. *In vitro* exposure to fetal calf serum can induce SSEA4 expression on the CB-HSC plasma membrane^29^. Thus, this finding weakens the physiological relevance of SSEA4 and its reliability as a MSC marker.

Our more relevant result concerned CD271. A high number of CD271-positive cells were found in BMMSC as well as in ADMSC, the other adult compartment analyzed, whereas fetal MSC showed very low or absent CD271 expression. The added value of our study is to comprehensively include MSC from many different sources, compared to the existing literature which focused mainly on one or two MSC types. For instance, many reports proposed CD271 as a marker of a bone marrow MSC subpopulation with distinct stemness and differentiation properties^30–37^. Other studies showed CD271 expression in ADMSC consistent with our data^38–41^. Furthermore, absent or very low CD271 expression in fetal MSC was observed by few groups^39,42^. In contrast with previous studies^31,43^, we did not observe low CD90 and CD73 expression in the CD271^+^ adult MSC fraction.

Intriguingly, flow cytometry data provided sufficient information as to clearly separate MSC based on the tissue of origin by clustering and principal component bioinformatics analyses. Concerning the hierarchical clustering, PVC and WJMSC showed very similar features, consistent with their shared umbilical cord tissue niche. ADMSC were closer to HSF than to other MSC. This finding could hint at a lower degree of multipotency or more committed state of ADMSC, which might resemble differentiated stroma rather than a progenitor cell. Among the fetal MSC, AFC seemed to possess the most unique characteristics, as highlighted by their independent clustering compared to other fetal MSC. This aspect is in line with the peculiar tissue source of these cells (amniotic fluid) and the very early gestational age for harvest^44^. Principal component analysis reinforced the concept of distinct immunophenotypes for fetal and adult MSC, with the latter showing higher heterogeneity. Our laboratory previously explored the notion that MSC retain memory of their tissue of origin at the RNA level^45^. Specifically, we demonstrated that, in the context of a mainly shared miRNome composition, BMMSC showed privileged expression of bone marrow niche-related miRNAs. Compared to CBMSC, ADMSC revealed a molecular pattern highly compatible with an epithelial-like phenotype. Furthermore, we demonstrated that the adipogenic differentiation potential of MSC is strictly dependent on their adult or fetal tissue harvest^46^. Also in this work we observed relevant correlation of surface marker protein expression to respective messenger RNA levels.

As already discussed previously about SSEA4, the expression of other surface markers of *in vitro*-cultured MSC could be biased by the culture conditions. For instance, the use of undefined supplements, such as fetal serum or platelet lysate, may determine the acquisition of specific immunophenotypic traits^47^. Thus, notwithstanding the robust results obtained in our experimental setting, we performed a mandatory validation of the antigens of interest on MSC that were isolated and expanded by an independent group, following their culture conditions. We observed that NG2 was markedly biased by laboratory-specific experimental conditions. By contrast, the CD271-positive subpopulation was a feature of adult MSC, variably affected by the culture conditions applied. These results indicate that careful attention and validation must be applied to cell surface markers, as they could be generated by *in vitro* artifacts.

As the main conclusion of the present study, we contributed to phenotype-function correlation studies showing that a CD271^+^ subpopulation is present in adult MSC as compared to fetal MSC, comparing MSC isolated from many tissues. This subpopulation can be found at higher percentages soon after isolation under serum-based culture conditions, and contribute to heterogeneous adult MSC properties. Precise cell identity definition is crucial in the context of MSC clinical applications to choose the most appropriate therapeutic stem cell population, as requested by regulatory boards. Finally, immunophenotypic markers currently used in clinical studies appear to define only stromal, but not stem, MSC identity.

## Methods

### Preparation of stromal cell populations

Each stromal population type was isolated from eight independent healthy donors, as previously described for ADMSC, BMMSC^46^ and CBMSC (Long Living (LL-)CBMSC^9^), for AFC^48^, for PVC^49^, for WJMSC^50^. Three human skin fibroblasts (HSF) were also used^51^. Donors’ age was 55 ± 5 years for ADMSC, 33 ± 20 years for BMMSC, 48 ± 12 years for HSF, 38–42 gestational weeks for CBMSC, 16 gestational weeks for AFC, 30–33 gestational weeks for PVC, 38–42 gestational weeks for WJMSC. Informed consent was obtained and the study was approved by the Ethical Committee of Fondazione IRCCS Ca’ Granda Ospedale Maggiore Policlinico. All the experiments were performed according to the amended Declaration of Helsinki. All stromal populations, including external MSC samples (*n* = 2 ADMSC, *n* = 2 BMMSC, *n* = 2 WJMSC, *n* = 2 CBMSC), were cultured in αMEM-GlutaMAX (Thermo Fisher Scientific, Waltham, MA, USA) supplemented with 20% of the same fetal bovine serum batch (FBS, lot 1315149; Thermo Fisher Scientific). AFC were cultured in a specific medium for amniotic cells (Amniomed; Euroclone, Pero, Italy). MSC were also cultured in αMEM-GlutaMAX (Thermo Fisher Scientific) supplemented with 20% FBS (lot 42Q3065K; Thermo Fisher Scientific) or 5% human peripheral blood platelet lysate (PL), and in Prime-XV serum- and xeno-free chemically-defined good manufacturing practices (GMP)-grade MSC medium (91135; Irvine Scientific, Santa Ana, CA, USA) on 5 µg/cm^2^ fibronectin-coated surfaces (31002; Irvine Scientific). PL was a pool of thirty donors and was kindly provided by our GMP facility. Characterization of differentiation properties was previously performed by standard adipogenic, osteogenic and chondrogenic protocols^52^. PD-MSC were obtained after 2 weeks of culture with αMEM-GlutaMAX (Thermo Fisher Scientific) supplemented with 20% FBS (lot 42Q3065K; Thermo Fisher Scientific)^16^ of human (h)iPSC generated and characterized by uSTEM company (uSTEM, Padova, Italy) from BJ fibroblast cell line. The following crucial cultural parameters were maintained homogenous in all samples: confluence (80–90%), passage (3–5), a medium change 24 hours before cell harvesting or collection of conditioned media, same culture vessel. Moreover, cell culture maintenance was performed by a dedicated operator.

### CFU-F assay

Two hundred MSC were plated per 100-mm Petri dish in their culture medium. The medium was changed weekly and after two weeks the cells were washed with PBS, fixed with methanol and stained with carbolic gentian violet solution (Ral diagnostics, Cedex, France). After 2 washing steps with milliQ-grade water, colonies with diameter bigger than 1–2 mm were counted.

### Flow cytometry

To determine MSC and HSF immunophenotype, 100,000 cells were harvested for each stromal population. All cultures were analyzed at 70–80% confluence to avoid cell growth-related biases. The cells were centrifuged for 7 minutes at 350 × g, after which the supernatant was discarded. The cell pellet was then resuspended and incubated with fluorophore-conjugated antibodies in a total volume of 200 μL of PBS (Thermo Fisher Scientific) for 20 minutes in the dark at RT. The antibodies used and their combinations are specified in Supplementary Table 1. Isotype controls were used to assess autofluorescence. The samples were analyzed using a BD FACSCanto II cytometer and the BD FACSDiva analysis software (BD, Franklin Lakes, NJ, USA). Acquired events were plotted against forward and side scatter area physical parameters and homogenous cell populations were selected (P1 gate) excluding debris. At least 10,000 P1 events were acquired. Cell viability was measured by 7-AAD/Annexin V staining (BD); necrotic cells were <5% and apoptotic cells were <10% for all stromal populations. The markers of interest were then measured in histograms or dot plots by an analytical gate (P2) for marker-positive events determined such that it included less than 1% of P1-gated negative isotype-control events. Analytical data, including percentage of positive events and Mean Fluorescence Intensity (MFI) of P1-gated events were analyzed in Excel; MFI ratio was calculated as MFI (stained sample)/MFI (unstained sample); fold changes for percentages of marker-positive events were calculated comparing MSC to HSF as differentiated stromal control. The use of the application setting option for the BD cytometer parameter adjustment guaranteed consistent and unbiased analysis of samples within different analysis and experiments. Finally, to validate surface markers on MSC samples generated by an independent laboratory, the following antibodies were used following the gating strategy described above: AF647-conjugated mouse anti-human CD271 antibody (560877; Becton Dickinson, Franklin Lakes, NJ, USA), PE-conjugated mouse anti-human NG2 antibody (IM3454U; Beckman Coulter, Bea, CA, USA).

### RNA extraction

Total RNA was isolated using RNeasy Plus Mini-kit (Qiagen, Hilden, Germany), following manufacturer’s instructions. RNA purity was determined by spectrophotometric analysis with A_260_/A_230_ ratio and A_260_/A_280_ ratio higher than 2.1 and 2.0, respectively. RNA quality and integrity was confirmed by agarose gel electrophoresis, which showed absence of ribosomal RNA degradation with a 28 S/18 S rRNA amount ratio around 2 for all samples.

### Real-time qRT-PCR

First strand cDNA was synthesized from 800 ng of total RNA in a 20 μL final volume reaction, using the iScript cDNA synthesis kit (Bio-Rad Laboratories, Hercules, CA, USA), according to the manufacturer’s instructions. Primer pairs were designed in-house using the NCBI Primer Designing Tool (http://www.ncbi.nlm.nih.gov/tools/primer-blast/), selecting only the primers spanning an exon-exon junction and producing a PCR amplificate with a length between 70 and 150 base pairs. Real-time qRT-PCR was carried out using SsoFast EvaGreen Supermix (Bio-Rad Laboratories) in the CFX96 real-time PCR Detection System instrument (Bio-Rad Laboratories), using standard PCR conditions. All experiments were performed in triplicates and values showing >0.5 Ct variation compared to triplicate means were discarded. Each assay included a blank. To confirm product specificity, a melting curve analysis was performed after each amplification. Relative gene expression was normalized to *ACTB*, *B2M*, *GAPDH*, and *RPLP0* expression using the ΔΔC t method. For statistical analysis and expression data generation, the Bio-Rad CFX Manager software was used. Primer sequences will be provided upon request.

### Sorting

Cell sorting of CD271-positive cells was performed as follows. Two millions of cells were stained with an AF647 labeled mouse anti human CD271 (BD) for 20 minutes at room temperature. Cells were then washed twice with PBS 2% FBS (Thermo Fisher Scientific) and filtered with a 70 µm sterile filter. CD271^+^ cells were separated using a BD FACSAria III cell sorter (BD) with an overall purity >97%. The collected cells were used for further analyses.

### Osteogenic differentiation

Osteogenic differentiation of sorted adult MSC was induced as previously described^9,10^. Briefly, cell cultures at 80% confluence were incubated for three weeks in osteogenic differentiation medium (Lonza, Basel, Switzerland). Differentiation was confirmed by staining with Alizarin Red S (cat. no. 130-22-3; Sigma-Aldrich, Saint Louis, MI, USA).

### Statistical analysis

All statistical analyses were performed with Prism software (Graphpad, La Jolla, CA, USA). The statistical tests used and sample sizes are specified for each experiment in the figure legends. Briefly, normality of the datasets was addressed by Kolmogorov–Smirnov test and followed by *t*-test to demonstrate statistically significant differences between two experimental groups. Alternatively, nonparametric Mann–Whitney–Wilcoxon test was applied. Outliers of Real-time qRT-PCR datasets were identified by ROUT method (Q = 1%). The differences were considered significant with the following *P*-values (*P*): *P < 0.05; **P < 0.01; ***P < 0.001; ****P < 0.0001. Flow cytometry data were clustered using R software online (Wessa P, 2017, Hierarchical Clustering (v1.0.3) in Free Statistics Software (v1.1.23-r7); http://www.wessa.net/rwasp_hierarchicalclustering.wasp/). PCA was performed using Multibase 2015 add-in software for Excel (http://www.numericaldynamics.com/).

### Data availability

All data will be available upon request.

## Electronic supplementary material

Supplementary information
