# Supplementary material for: Low-affinity Nerve Growth Factor Receptor (CD271) Heterogeneous Expression in Adult and Fetal Mesenchymal Stromal Cells
Source: Sci Rep. 2018 Jun 18;8:9321. doi: 10.1038/s41598-018-27587-8 (PMC6006357; doi:10.1038/s41598-018-27587-8)
Supplement: Supplementary file 1 — Supplementary information [file 41598_2018_27587_MOESM1_ESM.pdf]

Low-affinity Nerve Growth Factor Receptor (CD271) Heterogeneous Expression in Adult and Fetal Mesenchymal Stromal Cells

[1]Mario Barilani, [1]Federica Banfi, [1]Silvia Sironi, [1]Enrico Ragni, [2,3]Salomé Guillaumin, [1]Francesca Polveraccio, [4,5]Lorenzo Rosso, [6]Monica Moro, [7]Giuseppe Astori, [8,9]Michela Pozzobon and [1,*]Lorenza Lazzari

[1]Laboratory of Regenerative Medicine – Cell Factory, Fondazione IRCCS Ca' Granda Ospedale Maggiore Policlinico, 20122, Milano, Italy

[2]Regenerative, Modular & Developmental Engineering Laboratory (REMODEL), Biomedical Sciences Building, National University of Ireland Galway (NUI Galway), H91 CF50, Galway, Ireland

[3]Science Foundation Ireland (SFI) Centre for Research in Medical Devices (CÚRAM), Biomedical Sciences Building, National University of Ireland Galway (NUI Galway), H91 CF50, Galway, Ireland

[4]Thoracic surgery and lung transplantation Unit, Fondazione IRCCS Ca' Granda Ospedale Maggiore Policlinico, 20122, Milano, Italy

[5]University of Milan, 20122, Milano, Italy

[6]INGM, National Institute of Molecular Genetics "Romeo ed Enrica Invernizzi", 20122, Milan, Italy

[7]Advanced Cellular Therapy Laboratory - Hematology Unit, S. Bortolo Hospital - ULSS 6, Contra' San Francesco 41, 36100, Vicenza, Italy

[8]Stem Cells and Regenerative Medicine Lab., Women's and Children's Health Dept., University of Padova, Via Giustiniani 3, 35128, Padova, Italy.

[9]Foundation Institute of Pediatric Research "Città della Speranza", Corso Stati Uniti 4, 35127, Padova, Italy.

*Corresponding author (lead author)

Lorenza Lazzari, PhD

Laboratory of Regenerative Medicine – Cell Factory

Fondazione IRCCS Ca’ Granda Ospedale Maggiore Policlinico

Via F. Sforza 35, 20122, Milano, Italy

Phone: +39 02 5503 4053; Fax: +39 02 5503 2796

Email: lorenza.lazzari@policlinico.mi.it

Supplementary table 1

Tube 1	Fluorochrome	Code	Company
Annexin V	FITC	556419	Becton Dickinson, Franklin Lakes, NJ, USA
Mouse anti-human CD271	PE	557196	Becton Dickinson, Franklin Lakes, NJ, USA
7-AAD	/	559925	Becton Dickinson, Franklin Lakes, NJ, USA
Tube 2			
Mouse anti-human PDGFRβ	PE	FAB1263P	R&D Systems, Minneapolis, MN, USA
Mouse anti-human CD146	PE-Cyanine 7	562135	Becton Dickinson, Franklin Lakes, NJ, USA
Mouse anti-human CD56	PE-Cyanine 5	A07789	Beckman Coulter, Bea, CA, USA
Tube 3			
Mouse anti-human SSEA4	FITC	560126	Becton Dickinson, Franklin Lakes, NJ, USA
Mouse anti-human NG2	PE	IM3454U	Beckman Coulter, Bea, CA, USA
Mouse anti-human CD34	PerCP-Cyanine 5.5	347222	Becton Dickinson, Franklin Lakes, NJ, USA
Tube 4			
Mouse anti-human CD90	PE	561970	Becton Dickinson, Franklin Lakes, NJ, USA
Mouse anti-human CD105	PerCP-Cyanine 5.5	560819	Becton Dickinson, Franklin Lakes, NJ, USA
Mouse anti-human CD73	PE-Cyanine 7	561258	Becton Dickinson, Franklin Lakes, NJ, USA
Mouse anti-human CD271	AlexaFluor 647	560877	Becton Dickinson, Franklin Lakes, NJ, USA
Tube 5			
Mouse anti-human CD34	FITC	560942	Becton Dickinson, Franklin Lakes, NJ, USA
Mouse anti-human CD14	PerCP-Cyanine 5.5	562692	Becton Dickinson, Franklin Lakes, NJ, USA
Mouse anti-human CD45	APC-H7	560274	Becton Dickinson, Franklin Lakes, NJ, USA

Supplementary table 1. Flow cytometry experimental layout.

Supplementary figure 1

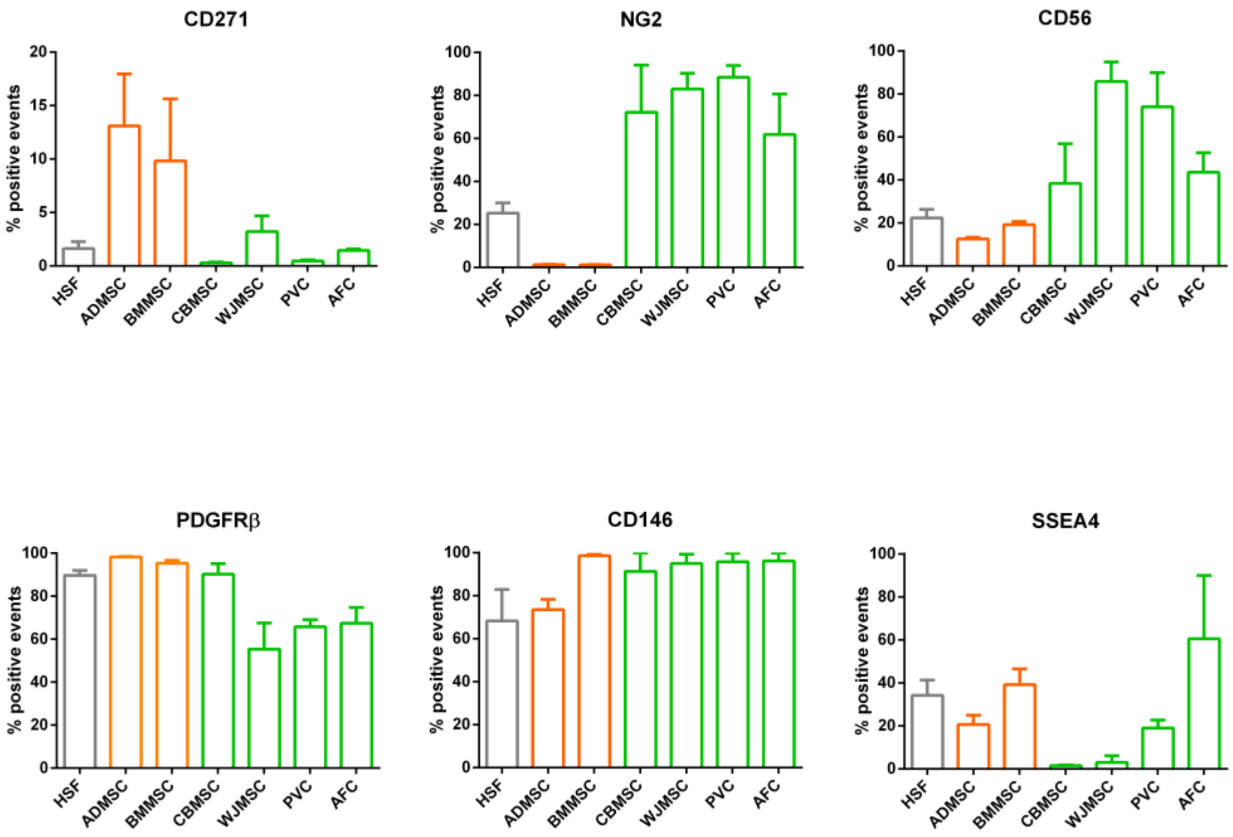

Supplementary figure 1. Surface marker expression percentages. The histograms show the percentages of surface marker expression (% positive events) for all the cell types analyzed by flow cytometry. HSF, human skin fibroblasts; ADMSC, adipose tissue mesenchymal stem cells; BMMSC, bone marrow mesenchymal stem cells; CBMSC, cord blood mesenchymal stem cells; WJMSC, Wharton's jelly mesenchymal stem cells; PVC, perivascular cells; AFC, amniotic fluid cells.
